# Screening and addressing social needs of children and families enrolled in a pediatric weight management program: a protocol for a pilot randomized controlled trial

**DOI:** 10.1186/s40814-022-01080-6

**Published:** 2022-06-18

**Authors:** Gita Wahi, Stacey Marjerrison, Carline Gutierrez, Kimberley Krasevich, Katherine M. Morrison, Lehana Thabane

**Affiliations:** 1 Department of Pediatrics, McMaster University, Hamilton, Ontario Canada; 2 Children’s Exercise and Nutrition Centre, McMaster Children’s Hospital, Hamilton, Canada; 3 Department of Health Research Methods, Evidence, and Impact, McMaster University, Hamilton, Ontario Canada; 4 Pediatric Hematology and Oncology, McMaster Children’s Hospital, Hamilton, Ontario Canada; 5 School of Nursing, McMaster University, Hamilton, Ontario Canada; 6 Biostatistics Unit, St Joseph’s Healthcare Hamilton, Hamilton, Ontario Canada; 7 Faculty of Health Sciences, University of Johannesburg, Johannesburg, South Africa

**Keywords:** Pediatric obesity, Social needs, Screening

## Abstract

**Background:**

There is a paucity of evidence to support interventions that address the social needs of children and families with chronic medical conditions. The primary objective of this pilot randomized controlled trial (RCT) is to assess the feasibility of an intervention that screens for and addresses the social needs of children and families enrolled in a pediatric weight management clinic.

**Method:**

We will conduct a single-center, pilot RCT of 40 families with children enrolled in a pediatric weight management program at a tertiary children’s hospital in Ontario, Canada. Families who are experiencing unmet social needs will be randomized to either a community navigator or self-navigation of community resources. The primary feasibility outcomes and criteria for success include the following: (1) recruitment rates, will be successful if 80% of our target sample is met in the 6 months of recruitment; (2) uptake of intervention, will be considered successful if > 80% of families complete the intervention; and (3) follow-up of participants, will be considered successful if > 90% of participants complete all the study visits. The secondary outcomes include estimating the preliminary effects on body mass index, body composition, and quality of life at 6 months. The analysis of feasibility outcomes will be based on descriptive statistics, and analysis of secondary clinical outcomes will be reported as estimates of effect. We will not perform tests of significance since these analyses are purely exploratory.

**Discussion:**

This study is important because it will aim to improve the treatment of pediatric obesity by testing the feasibility of an intervention that addresses unmet social needs.

**Trial registration:**

ClinicalTrias.gov: NCT04711707 (Registered January 13, 2021).

## Background

One of the largest public health concerns for children in the last 40 years is the rising prevalence of overweight and obesity [1]. The urgency of this global concern is directly related to the complications of childhood obesity which are far-reaching and include, but are not limited to, childhood onset cardiometabolic sequalae including type 2 diabetes, hypertension, and dyslipidemia, as well as experiences of bullying, poor mental health, and eventual reduced life expectancy [2, 3]. Notably, the risk of childhood overweight/obesity is not shared evenly across the population. In a Canadian population-based study, 24% of children in high-income neighborhoods were overweight/obese compared with 35% of children living in low-income neighborhoods (odd ratio (OR) 1.29, 95% confidence intervals 1.14, 1.46) [4]. There are also major disparities in pediatric obesity treatment. In a systematic review of the impact of socioeconomic status on pediatric weight management programs, children with obesity from lower socio-economic neighborhoods had higher attrition rates and lower adherence to weight management programs than children from higher socio-economic neighborboods [5]. In a cohort of Canadian children enrolled in a weight management program between 2013 and 2017 (*n* = 847), 28% reported a low household income [6], which is higher than data from 2017 of 10% of Canadian children living in households experiencing poverty [7]. The observed inequities in the prevalence and treatment of pediatric obesity can be considered within a social determinants of health (SDoH) framework, which suggests that social and economic factors work upstream to influence the health of populations [8]. The SDoH are the “conditions in which people are born, grow, live, work, and age,” [3] and although the SDoH are important determinants of health outcomes, addressing social needs is not always screened for and addressed within clinical settings [9].

In a 2016 policy statement, the American Academy of Pediatrics called for early identification of social risks and advocated for connection to community supports [10]. There are recent systematic reviews by Gottlieb et al. of interventions addressing social needs in clinical settings and by Eder et al. addressing social risk screening and interventions connected to healthcare systems [11, 12]. Both reviews concluded that there is a paucity of evidence evaluating the impact of addressing unmet social needs on health outcomes and called for more high-quality evidence in this area [11, 12]. There is also limited evidence for screening and addressing social needs within pediatric weight management programs. An intervention study that addressed food insecurity among children treated for obesity observed 24% of patients had food insecurity however, a low proportion of those eligible enrolled in food subsidy programs (8%) [13]. We propose to build a robust body of evidence and identify efficacious interventions that address social needs and have a significant impact on important child health outcomes, specifically pediatric obesity.

Interventions to screen and address social needs in pediatric settings have most commonly been studied in primary care settings, with evidence of improved health outcomes with social needs navigation [14, 15]. However, pediatricians providing care to children with chronic medical conditions may have a unique opportunity to intervene and improve health outcomes [2, 3]. This opportunity is reinforced by the availability of tools to screen for social risk among pediatric patients and families [2, 7], local resources that provide community-level information to help guide clinician referrals to community resources [3, 16, 17], and existing models of successful navigation interventions in pediatric settings [14, 15]. The objectives of this pilot RCT are to assess the feasibility of a social needs screening intervention in a pediatric weight management clinic by evaluating recruitment strategies, uptake, and acceptability of the intervention.

## Methods

### Study design

We are conducting a single-center, blinded, pilot RCT. Families are screened for unmet social needs; those that screened positive are randomized into two arms, either the intervention with a community navigator or the control with self-navigation of resources. The trial is guided by the SPIRIT (Standard Protocol Items: Recommendations for Interventional Trials) checklist (see Appendix 1) [18]. Table 1 outlines the timeline of enrollment, intervention, and data collection.

**Table 1:**
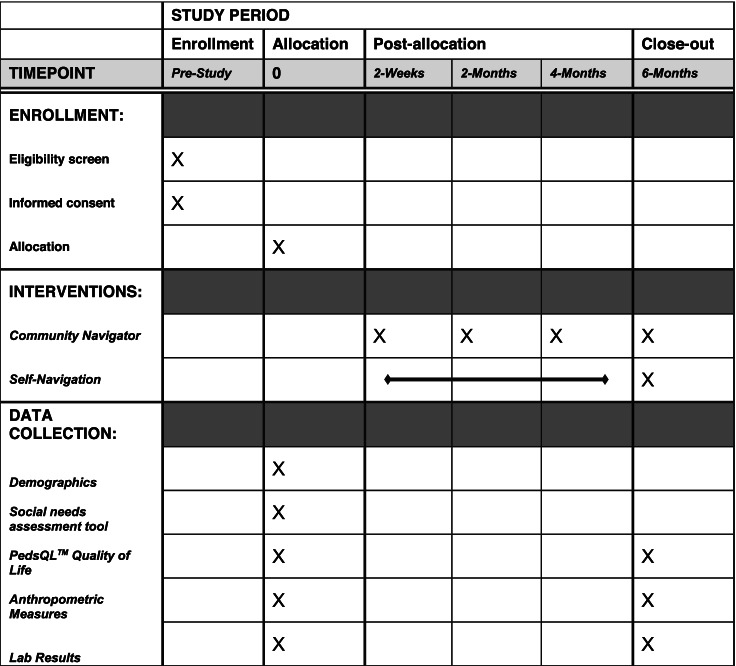
Schedule of enrollment, interventions, and data collection

### Setting

This study is conducted within the Growing Healthy Weight Management clinic in the Children’s Exercise and Nutrition Centre (CENC) at the McMaster Children’s Hospital in Hamilton, Ontario, Canada. The comprehensive pediatric weight management clinic is staffed by a pediatrician, endocrinologists, nurse practitioners, dieticians, exercise physiologist, kinesiologist, and psychologist.

### Study population

The following criteria must be met to be eligible to be enrolled in the study:

#### Inclusion criteria

1. Enrolled < 18 months in the CENC Growing Healthy Weight Management clinic
2. Child age between 2 and 18 years

#### Exclusion criteria

1. Child in the care of child protection services and/or living in group or foster care as children in these settings will not be living within typical family systems to have social needs addressed by this intervention.
2. Parents/guardians who cannot read and write in English

Of note, if a family was enrolled with one child and a subsequent child joins the clinic, the family is not eligible to re-enroll into the study.

### Sample size

The sample size for pilot studies can vary [19]. The sample size for this pilot study is based on feasibility considerations and will be 40 families randomized over 6 months. The CENC weight management program typically enrolls 200 patients per year, averaging 18 new patients per month. It is estimated that 80 families will need to be screened to reach our goal of 40 families randomized to the study: 20 in each of the two parallel arms. In previous unpublished pilot work, we observed that ~60% of families who enrolled in the CENC program lived in neighborhoods with high material deprivation as defined by the Ontario Marginalization Index [20]. Participants will be recruited over a 6-month period, 13 to 14 families per month. This pilot data will inform the sample size calculation for the main trial.

### Screening and recruitment strategy

Eligible families will be asked during a clinic visit by a clinician in their circle of care for consent to be contacted by phone or email. The names of those who consent will be recorded on a master list stored on a secure electronic server. E-mail addresses will be recorded for those who agree to this mode of communication. The research assistant (RA) will consult the master list to obtain a list of names and contact information for those interested in hearing more about the study. If they have agreed to be contacted, the parent or guardian will be contacted by the RA in advance of their next appointment to have the study protocol described to them and ask them if they are willing to consent to participate.

### Consent and assent

After verbal consent has been obtained over the phone, consent will be formalized using one of three methods. The various methods are being made available to accommodate COVID-19 restrictions, family preference, and Hamilton Integrated Research Ethics Board regulations: (1) families with the preference and access to email will be sent a REDCap link to provide digital consent/assent. The consent will be the first document to appear in REDCap with a series of yes/no questions. The questions will confirm that the study and their involvement are understood, all questions have been answered, and they are agreeing to participate. (2) Those without email access will be asked permission for us to mail documents for signed consent. These first two methods will be particularly important to employ when the patients are scheduled to be seen remotely. (3) And in the third option, when the patient has an in-person clinic appointment and has not previously enrolled using REDCap or mail-in, a member of the research team will join them at the clinic to answer questions and obtain signed consent and/or assent.

Parents or guardians will be approached if the child is under 8 years of age. For those between the ages of 8 and 15 (inclusive), the study will be described to both the parent or guardian and the child. The child will be asked to provide assent, and the parents or guardians will provide consent. If the child is over the age of 15, the child themselves can provide consent for the study team to have access to their clinical data. The parent or guardian will still be asked for consent to complete parent-directed questionnaires, regardless of the age of the child or youth enrolled in the clinic.

### Data collection and baseline measurements

Initial data collection includes demographic information, the social needs assessment tool, and a pediatric quality of life questionnaire [17–20]. The social needs assessment tool will be composed of a questionnaire based on a social history tool with the mnemonic “ITHELLPS,” which assesses for social risks and is adapted for a Canadian context, addressing housing needs, income and food insecurity, parental literacy, and transportation concerns [2, 21]. The tool was chosen given its development for a Canadian context and ease of use. Participants who reply “yes” to any of the social needs assessment tool questions will be randomized to either the intervention or control arm. Baseline demographic data, at the time of enrollment into the study, will include child’s age, sex, ethnicity, medical history (e.g., presence of chronic medical conditions), family structure, and socio-economic status. Socioeconomic status will be acquired from self-reported annual family household income, parental employment status, and parental education.

Data that will be collected from the chart will include child’s height, weight, blood pressure, percentage body fat, and body mass index z-score (zBMI). Lab work done during regular clinical care in the CENC will be collected including fasting glucose, lipids, liver enzymes, complete blood count, and ferritin. Recording of anthropometrics and lab work and quality-of-life questionaires will be repeated 6 months after initial enrollment.

### Trial intervention

All families who consent to participate in the study will complete the social needs assessment tool. Those who do not screen positive in any area of social need will not be randomized but will be included in the study for the purpose of comparison and will have follow-up data collected at 6 months. Those who screen positive in at least one social need will be randomized to receive one of two parallel interventions, community navigator (intervention) or self-navigation (control). The community navigator intervention will include an in-person, phone call, or videoconference visit (determined by participant preference) with a community navigator to help connect with appropriate services for their specific needs and geographic region of residence. Referrals will be sourced using the www.211ontario.ca website and tools as well as a regional services resource guide developed by the study team. The intervention will include bimonthly check-ins in person during clinic, over the phone, email, or videoconference. The self-navigation group will be given a paper or emailed an electronic copy of community resources and services. If there are any concerns from the social needs assessment tool, the RA will bring them to the attention of the PI who will discuss it with the clinical team to decide on the appropriate action to be taken.

### Allocation

Families who screen positive on the social needs assessment tool will be randomized to the community navigator or self-navigation arms using an allocation ratio of 1:1 with random permuted blocks of varying size of 2, 4, and 6. An advantage of block randomization is that group sizes are similar at the end of each block. Random block size will help to ensure that investigators or outcome assessors will not be able to decipher the block size and anticipate future allocations. Allocation concealment is the process that prevents any trial participant or investigator from knowing in advance the treatment to which subjects will be assigned and seeks to prevent selection bias [22]. In this study, allocation concealment will be achieved by using a central randomization system set up in REDCap.

### Blinding

Data analysts will be blind to the group allocation. Group allocation will be concealed until the final data analysis is performed. Families, research staff, and clinical staff will not be blinded to the group allocation.

### Outcomes

This pilot RCT will evaluate the feasibility of implementation and delivery of the intervention. We will consider it to be successful if we achieve the following primary outcomes:

1. Recruitment rates: Recruitment will be successful if 80% of our target sample is met in the 6 months of recruitment.
2. Uptake of intervention: Will be considered successful if > 80% of families complete the intervention.
3. Follow-up of participants: Will be considered successful if > 90% of families complete all the study visits.

Although not the focus of the pilot trial, we will collect the following secondary outcomes to test and refine the data collection process for a full-scale trial; the following measures will be collected at baseline visit at enrollment and then at 6 months:

1. Change in body mass index z-score (zBMI): This will be calculated using WHO growth charts, for age and sex. Height and weight of the child will be collected from the chart at baseline and at the end of the intervention. BMI will be calculated by dividing weight in kilograms by the square of the body height in meters squared.
2. Change in body composition: Body fat will be assessed at baseline and at the end of the intervention using the Quantum II BIA analyzer (RJL Systems). Bioelectrical impedance analysis (BIA) is non-invasive and portable.
3. Change in quality of life: We will measure health-related quality of life using the PedsQL™. Both the patient and the parent or guardian will be asked to complete the PedsQL™.

### Analysis and reporting

Participant characteristics will be reported using descriptive statistics. An intention to treat analysis will be used. The analysis of feasibility outcomes will be based on descriptive statistics reported as percentage (95% confidence interval [CI]), and these will be evaluated against the set criteria for success of feasibility. Analysis of secondary clinical outcomes will be reported as estimates of effect (95% CI). We will not perform tests of significance since these analyses are purely exploratory. All analyses will be formed using SPSS. Table 2 provides a summary of the objectives, corresponding outcomes, criteria for success of feasibility or hypotheses, and method of analysis.

**Table 2:** Summary of the objectives, outcomes, criteria for success of feasibility or hypotheses, and method of analysis

Aim	Objective	Outcome	Criteria for success of feasibility/hypothesis	Method of analysis
Primary	Feasibility of implementation and delivery of intervention	Recruitment rate	80% of target sample reached in 6 months	Descriptive, percentage (95% confidence interval [CI])
		Uptake of intervention	> 80% of participants recruited complete the intervention	Descriptive, percentage (95% confidence interval [CI])
		Follow-up	> 90% of participants complete all study visits	Descriptive, percentage (95% confidence interval [CI])
Secondary	Health outcomes	Change in zBMI	Intervention group will show improvement	Estimates of effect (95% CI)
		Change in body composition		Estimates of effect (95% CI)
		Change in quality of life		Estimates of effect (95% CI)

## Discussion

This trial is critical to understand if it is feasible to recruit and complete an intervention focused on screening for and addressing social needs in a pediatric weight management clinic. This work is also an important first step to determining the impact and feasibility of integrating the screening of social needs into the care of children with complex medical illnesses.

Although we initially developed this trial during the pre-pandemic era, initiating this study in the context of the global COVID-19 pandemic uncovered several important considerations and adaptations. First, the COVID-19 pandemic highlighted the significant disparities in health outcomes of those with unmet social needs. Throughout the COVID-19 pandemic, children have endured disruptions in academic, family, and social interactions [23]. Parents and caregivers have faced precarious employment, economic uncertainties, increased caregiver duties, and changes in work-life responsibilities [24]. Cumulatively, these changes have led to major and pervasive stressors on the family unit, which increases the urgency and need for this pilot RCT.

Since this study was initiated during the COVID-19 pandemic, we amended the original protocol to conduct the trial “remotely,” i.e., over phone, email, or videoconference with no in-person visits. The advantage of this change in the protocol is that it allows for ongoing recruitment despite changes in public health social distancing recommendations. However, this method of data collection also presents a series of challenges in our early experiences to date. For example, for the first five patients enrolled into the study, the time between consent to contact being obtained and verbal consent by phone ranged from 0 to 20 days with a mean value of 5.7 days. It took another 0 to 73 days to obtain digital consent and the completed surveys with a mean value of 9.6 days. With the extended response time, the RA spent more time following up with the potential participants to get their enrollment documents. Furthermore, there were five families who provided consent to contact in the clinic who could subsequently not be reached by telephone and another six who did not complete the consent and/or surveys after providing verbal consent. We postulate that if the study was conducted with research staff in the clinic, written consent and survey completion would all be done more efficiently. As the pandemic impacts continue to be felt in clinical research, understanding that we need to build more time for enrollment than we would have in the pre-pandemic era is an important consideration moving forward.

A further limitation of this study is the exclusion of families who have limited English proficiency (LEP). However, families with LEP may be at the most risk of navigating medical and social systems and therefore experiencing adverse health outcomes [25, 26]. Given these considerations, future work informed from this pilot RCT of a social needs intervention will be inclusive of families with LEP.

This study is important because it will aim to enhance the treatment of pediatric obesity, a common and chronic health concern, and has the potential to improve health outcomes by testing the feasibility of an intervention that addresses family-identified social needs. A pilot study is necessary to understand if recruitment strategies and the implementation of the intervention are feasible in this clinical setting. The results will be used to design a larger study that has the potential to have a significant impact on the health of children.

## Data Availability

Not applicable

## Abbreviations

BIA: Bioelectrical impedance
CENC: Children’s Exercise and Nutrition Centre
OR: Odds ratio
RA: Research assistant
RCT: Randomized controlled trial
SDoH: Social determinants of health
SPIRIT: Standard Protocol Items: Recommendations for Interventional Trials
zBMI: Body mass index z-score
